# Exploratory Risk Assessment of Cardiovascular Diseases Using Antiphospholipid Antibody-Related Scores in Patients with ANCA-Associated Vasculitis

**DOI:** 10.3390/jcm15207925

**Published:** 2026-10-14

**Authors:** Oh Chan Kwon, Yong-Beom Park, Sang-Won Lee

**Affiliations:** 1 Division of Rheumatology, Department of Internal Medicine, Gangnam Severance Hospital, Yonsei University College of Medicine, Seoul 06273, Republic of Korea; ockwon@yuhs.ac; 2 Division of Rheumatology, Department of Internal Medicine, Yonsei University College of Medicine, Seoul 03722, Republic of Korea; yongbpark@yuhs.ac; 3 Institute for Immunology and Immunological Diseases, Yonsei University College of Medicine, Seoul 03722, Republic of Korea

**Keywords:** antineutrophil cytoplasmic antibody, vasculitis, antiphospholipid antibody, arterial thrombosis, risk

## Abstract

**Background:** Antiphospholipid antibodies have been found in patients with antineutrophil cytoplasmic antibody (ANCA)-associated vasculitis (AAV). This study aimed to evaluate the accuracy of the global antiphospholipid syndrome score (GAPSS) derivatives in predicting cardiovascular diseases in Korean patients with AAV, and to derive a simple candidate index for cardiovascular diseases. **Methods:** This was a retrospective cohort study including 170 patients with AAV. The adjusted GAPSS (aGAPSS), aGAPSS specific for cardiovascular diseases (aGAPSScvd), and aGAPSS for Korean patients with APS (aGAPSS [KAPS]) were calculated in each patient. The cardiovascular diseases were defined as cerebrovascular accident (CVA) or acute coronary syndrome (ACS). The accuracy of each index was assessed by estimating the area under the curve (AUC). An exploratory multivariable Cox proportional hazards model with a backward elimination method was performed to derive a candidate index. Covariates consisting the aGAPSScvd were included in the model. **Results:** The AUCs of the aGAPSS, aGAPSScvd, and aGAPSS (KAPS) for CVA were 0.507 (*p* = 0.926), 0.531 (*p* = 0.676), and 0.493 (*p* = 0.923), respectively. The AUCs of the aGAPSS, aGAPSScvd, and aGAPSS (KAPS) for ACS were 0.613 (*p* = 0.441), 0.752 (*p* = 0.086), and 0.796 (*p* = 0.043), respectively. The exploratory multivariable model revealed a candidate index, aGAPSScvd for ACS in Korean patients with AAV (aGAPSScvd [KAAV]) = 2 × lupus anticoagulant (LA) + 2 × obesity. The AUC of the aGAPSScvd (KAAV) in predicting ACS was 0.843 (*p* = 0.019). **Conclusions:** The two-item candidate index aGAPSScvd (KAAV), consisting of LA and obesity, showed discriminatory ability for ACS, which warrants further validation in larger studies.

## 1. Introduction

Antineutrophil cytoplasmic antibody (ANCA)-associated vasculitis (AAV) is a systemic vasculitis, mainly involving small- and medium-sized vessels [1]. Compared with age- and sex-matched controls, patients with AAV are at a higher risk of cardiovascular diseases [2]. Notably, cardiovascular diseases are the leading cause of death in patients with AAV [3], and thus deserve attention when treating patients with AAV. It is important to assess the risk of cardiovascular diseases in patients with AAV, which could possibly lead to stricter monitoring and earlier detection of cardiovascular diseases in high-risk patients.

Interestingly, studies have reported presence of antiphospholipid antibodies (aPLs) in patients with AAV [4,5]. Given that aPLs are closely related to thrombosis events [6], and that the main pathogenesis of cardiovascular diseases is arterial thrombosis [7], aPLs could be important factors that need to be considered when assessing the risk of cardiovascular diseases in patients with AAV. Although a previous study has developed a risk score (−3.9 + [0.04 × age] − [0.95 × proteinase 3 (PR3)-ANCA] + [0.68 × hypertension]) to predict cardiovascular diseases in patients with granulomatosis with polyangiitis (GPA) and microscopic polyangiitis (MPA), the presence of aPLs was not taken into account [8].

The global antiphospholipid syndrome score (GAPSS) is a scoring system that predicts thrombosis in patients with antiphospholipid syndrome (APS) by incorporating traditional cardiovascular risk factors and aPLs [9]. The scoring system consists of hyperlipidaemia, arterial hypertension, anticardiolipin (aCL) immunoglobulin (Ig) G/IgM, anti-β2 glycoprotein I (aβ2GPI) IgG/IgM, anti-phosphatidylserine/prothrombin (anti-PS/PT) IgG/IgM, and lupus anticoagulant (LA) [9]. There are some modified versions of the GAPSS. The adjusted GAPSS (aGAPSS) is a modified version of GAPSS that excludes anti-PS/PT from the scoring system as these tests are not widely used in clinical practice [10,11]. We previously assessed whether aGAPSS is associated with the risk of cardiovascular diseases (defined as cerebrovascular accidents [CVAs] and acute coronary syndrome [ACS]) in patients with AAV, but no significant association was observed between aGAPSS and cardiovascular diseases in patients with AAV [12].

However, there are other derivatives of GAPSS that have not been tested in patients with AAV. The aGAPSS specific for cardiovascular diseases (aGAPSScvd) is another derivative of GAPSS, which incorporates obesity, smoking habit and diabetes into the aGAPSS [13]. Considering that the risks of venous thrombosis and arterial thrombosis are different [14], the aGAPSScvd was developed to assess the risk of arterial thrombosis more specifically, and thereby the risk of cardiovascular diseases [13]. The aGAPSScvd was better in detecting cardiovascular diseases than aGAPSS in subjects with aPLs [13]. Moreover, we recently developed a new scoring system, the aGAPSS for Korean patients with APS (aGAPSS [KAPS]), which has a higher accuracy than aGAPSS in predicting thrombosis in Korean patients with APS [15]. Although aGAPSS has previously been evaluated in patients with AAV, the discriminatory performance of other GAPSS derivatives, including aGAPSScvd and aGAPSS (KAPS), for CVA and ACS in patients with AAV remains unknown.

In this study, we assessed the discriminatory performance of aGAPSS derivatives, including aGAPSScvd and aGAPSS (KAPS), for CVA and ACS in Korean patients with AAV. We also explored whether a simple candidate index for ACS could be derived from the components of aGAPSScvd.

## 2. Materials and Methods

### 2.1. Study Cohort

Medical records of 195 patients with AAV enrolled in the Severance Hospital ANCA-associated vasculitis cohort were retrospectively reviewed for inclusion. The inclusion criteria for the present study were as follows: (i) patients who were diagnosed with AAV for the first time at the Division of Rheumatology, the Department of Internal Medicine, Yonsei University College of Medicine Severance Hospital, from October 2000 to March 2023; (ii) patients who met the 2007 European Medicine Agency algorithm for AAV and polyarteritis nodosa, the revised 2012 Chapel Hill Consensus Conference Nomenclature of Vasculitides, and the 2022 American College of Rheumatology/European Alliance of Associations for Rheumatology classification criteria for MPA, GPA, and eosinophilic granulomatosis with polyangiitis (EGPA) [16,17,18,19,20,21]; (iii) patients who had no missing data to calculate the previously developed risk score [8], aGAPSS [10,11], aGAPSScvd [13], and aGAPSS (KAPS) [15] at AAV diagnosis; (iv) patients who had been followed up for three months or greater; (v) patients who had never been diagnosed with APS before or at AAV diagnosis; (vi) patients who had no concomitant serious medical conditions mimicking AAV such as infectious diseases requiring hospitalization or malignancies at AAV diagnosis; and (vii) patients who had not been exposed to immunosuppressive drugs or glucocorticoids (≥10 mg equivalent to prednisolone) within three months before AAV diagnosis. Co-existing serious medical conditions and immunosuppressive drugs administered were identified by the 10th revised International Classification Diseases and the Korean Drug Utilization Review system, respectively. Applying these inclusion criteria, a total of 170 patients with AAV were included for analysis. Although concomitant autoimmune or inflammatory diseases potentially associated with LA positivity were not systematically excluded, none of the included patients had Hashimoto’s thyroiditis, type 1 diabetes, inflammatory bowel disease, or chronic hepatitis B infection. Patients were followed up from the date of AAV diagnosis to the date of cardiovascular disease occurrence or the last hospital visit, whichever came first.

This study was approved by the Institutional Review Board (IRB) of Severance Hospital (Seoul, Republic of Korea, IRB No. 4-2016-0901). The requirement for additional written informed consent was waived by the IRB owing to the retrospective nature of this study and the use of anonymised patient data.

### 2.2. Covariates and Outcome

The following covariates at the time of AAV diagnosis were collected: age, sex, body mass index (BMI), smoking history, AAV subtype, positivity of myeloperoxidase (MPO)-ANCA, PR3-ANCA, aCL IgG/IgM, aβ2GPI IgG/IgM, and LA, and presence of hyperlipidaemia, arterial hypertension, type 2 diabetes mellitus (T2DM), and obesity (BMI ≥ 25 kg/m^2^ according to the Korean Society for the Study of Obesity criteria [22]). aPL positivity was confirmed by repeat testing at a 3-month interval. Other thrombophilic factors, including inherited thrombophilia, were not assessed in the present study. The following medications used during the follow-up period were collected: glucocorticoids, cyclophosphamide, rituximab, azathioprine, mycophenolate mofetil, tacrolimus, and methotrexate. The outcomes were cardiovascular diseases defined as CVA and ACS, diagnosed based on imaging studies.

### 2.3. Calculation of the Previous Risk Score, aGAPSS, aGAPSScvd, and aGAPSS (KAPS)

The previous risk score was calculated as −3.9 + (0.04 × age) − (0.95 × PR3-ANCA) + (0.68 × hypertension) [8]. The aGAPSS was calculated as 5 × aCL IgG/IgM + 4 × aβ2GPI IgG/IgM + 4 × LA + 3 × hyperlipidaemia + 1 × arterial hypertension [10,11]. The aGAPSScvd was calculated as 5 × aCL IgG/IgM + 4 × aβ2GPI IgG/IgM + 4 × LA + 3 × hyperlipidaemia + 1 × arterial hypertension + 2 × obesity + 2 × T2DM + 1 × smoking [13]. The aGAPSS (KAPS) was calculated as 1 × T2DM + 2 × LA + 1 × aβ2GPI [15].

### 2.4. Statistical Analysis

We summarized the patients’ characteristics using descriptive analysis. Continuous variables were expressed as median (Q1–Q3) and categorical variables as number (%). The discriminatory performance of the previous risk score, aGAPSS, aGAPSScvd, and aGAPSS (KAPS) for CVA and ACS was explored using receiver operating characteristic (ROC) curve analysis, and areas under the curves (AUCs) with 95% confidence intervals (CIs) were estimated. For ACS, an exploratory multivariable Cox proportional hazards model with backward elimination was fitted using the components of aGAPSScvd as candidate covariates. Covariates with *p* > 0.2 were eliminated at each step. A relatively liberal elimination threshold of *p* > 0.2 was used to avoid prematurely excluding potentially informative variables in this exploratory analysis. A candidate index was derived from the β coefficients of the variables remaining in the final model, which were rounded to the nearest integer. Its discriminatory performance was assessed using ROC analysis. Internal validation of the candidate index was performed using 1000 nonparametric bootstrap resamples. The complete model-development procedure, including variable selection and derivation of the candidate index, was repeated in each bootstrap sample. Optimism was estimated as the mean difference between the AUC obtained in each bootstrap sample and that obtained when the corresponding bootstrap-derived model was applied to the original cohort. The optimism-corrected AUC was calculated by subtracting the estimated optimism from the apparent AUC. A data-driven cut-off was selected using the maximum Youden index, and ACS-free survival was illustrated using Kaplan–Meier analysis with a log-rank comparison. Statistical significance was set at a *p* value of <0.05. All analyses were conducted using SPSS software (version 28.0; IBM Corporation, Armonk, NY, USA).

## 3. Results

### 3.1. Characteristics of the Study Population

The characteristics of the 170 patients with AAV are reported in Table 1. The median age was 63.0 (50.0–70.3) years and 39.4% were men. The median value of BMI was 22.7 (20.3–24.2) kg/m^2^, and 5.3% were smokers. MPA (54.7%) was the most common AAV subtype, followed by GPA (25.9%) and EGPA (19.4%). MPO-ANCA and PR3-ANCA were positive in 72.9% and 15.3% of the patients, respectively. Regarding the items of aGAPSScvd, aCL IgG/IGM, aβ2GPI IgG/IgM, LA, hyperlipidaemia, hypertension, T2DM, and obesity were present in 9.4%, 5.3%, 22.9%, 18.2%, 36.5%, 24.1%, and 19.4% of the patients, respectively. The median values of the previous risk score, aGAPSS, aGAPSScvd, and aGAPSS (KAPS) were −1.26 (−1.95–−0.93), 3.0 (0.0–4.0), 3.0 (0.0–6.0), and 0.0 (0.0–1.0), respectively. CVA occurred in 17 (10.0%) patients during a follow-up duration of 32.1 (10.6–59.2) months, and ACS occurred in four (2.4%) patients during a follow-up duration of 35.4 (12.4–61.2) months. Glucocorticoids were used in majority of the patients (98.8%). Cyclophosphamide (60.0%) was the most commonly used immunosuppressant.

At the time of ACS occurrence, the Birmingham Vasculitis Activity scores (BVASs) of the four patients were four, seven, four, and four, respectively. The corresponding organ manifestations contributing to the BVAS were cardiovascular involvement (ischemic cardiac pain), cardiovascular and pulmonary involvement (ischemic cardiac pain and a new lung nodule), cardiovascular involvement (ischemic cardiac pain), and cardiovascular involvement (ischemic cardiac pain), respectively. The corresponding erythrocyte sedimentation rates were 17, 41, 46, and 35 mm/h, C-reactive protein values were 5.8, 8.6, 9.5, and 9.1 mg/L, and estimated glomerular filtration rates were 69, 77, 46, and 49 mL/min/1.73 m^2^, respectively. The prednisolone-equivalent glucocorticoid doses at the time of ACS were 2.5, 5, 5, and 5 mg/day, respectively.

### 3.2. Accuracy of the Previous Risk Score, aGAPSS, aGAPSScvd, and aGAPSS (KAPS)

**Figure 1:**
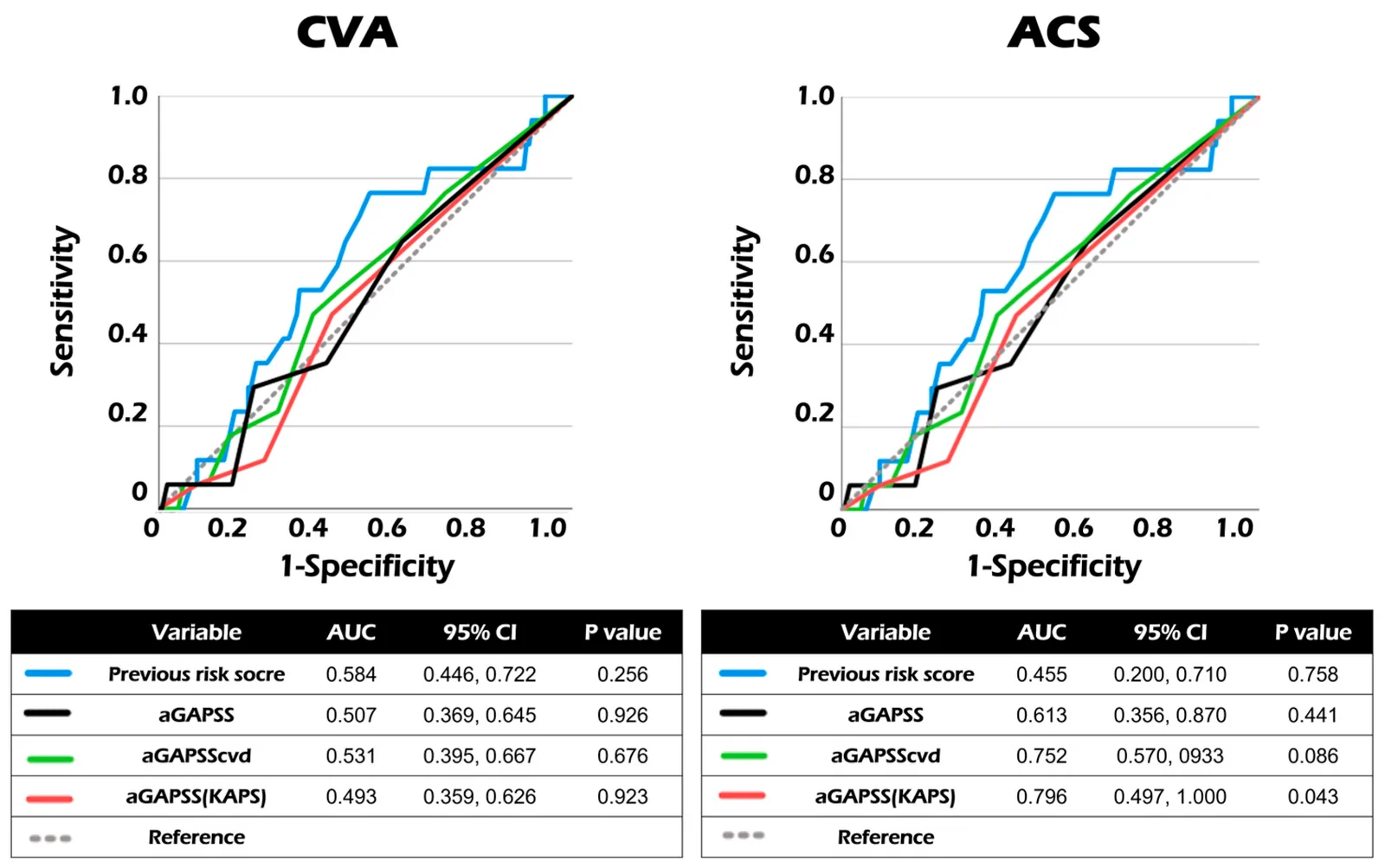
AUCs for predicting CVA and ACS. AUC: Area under the curve; CVA: cerebrovascular accident; ACS: acute coronary syndrome; CI: confidence interval; aGAPSS: adjusted global antiphospholipid syndrome score; aGAPSScvd: aGAPSS specific for cardiovascular diseases; aGAPSS (KAPS): aGAPSS for Korean patients with antiphospholipid syndrome.

The ROC curves of the previous risk score, aGAPSS, aGAPSScvd, and aGAPSS (KAPS) for predicting CVA and ACS, respectively, are shown in Figure 1. The AUCs of the previous risk score, aGAPSS, aGAPSScvd, and aGAPSS (KAPS) in predicting CVA were 0.584 (95% CI: 0.446–0.722, *p* = 0.256), 0.507 (95% CI: 0.369–0.645, *p* = 0.926), 0.531 (95% CI: 0.395–0.667, *p* = 0.676), and 0.493 (95% CI: 0.359–0.626, *p* = 0.923), respectively, with none reaching statistical significance. The AUCs of the previous risk score, aGAPSS, aGAPSScvd, and aGAPSS (KAPS) for ACS were 0.455 (95% CI: 0.200–0.710, *p* = 0.758), 0.613 (95% CI: 0.356–0.870, *p* = 0.441), 0.752 (95% CI: 0.570–0.933, *p* = 0.086), and 0.796 (95% CI: 0.497–1.000, *p* = 0.043), respectively. Except for the previous risk score, the AUCs of each scoring system were numerically larger for ACS than CVA. Remarkably, aGAPSS (KAPS) was statistically significant in predicting ACS.

### 3.3. Exploratory Derivation of a Candidate Index

Because the aGAPSS derivatives showed numerically greater discrimination for ACS than for CVA, an exploratory model was fitted for ACS. The results of the Cox proportional hazards model based on the components of aGAPSScvd are summarized in Table 2. In the final step, LA and obesity remained in the model. Using the rounded β coefficients, the following candidate index was derived: aGAPSScvd for ACS in Korean patients with AAV (aGAPSScvd [KAAV]) = 2 × LA (presence = 1, absence = 0) + 2 × obesity (presence = 1, absence = 0).

### 3.4. Discriminatory Performance of the Candidate Index

**Figure 2:**
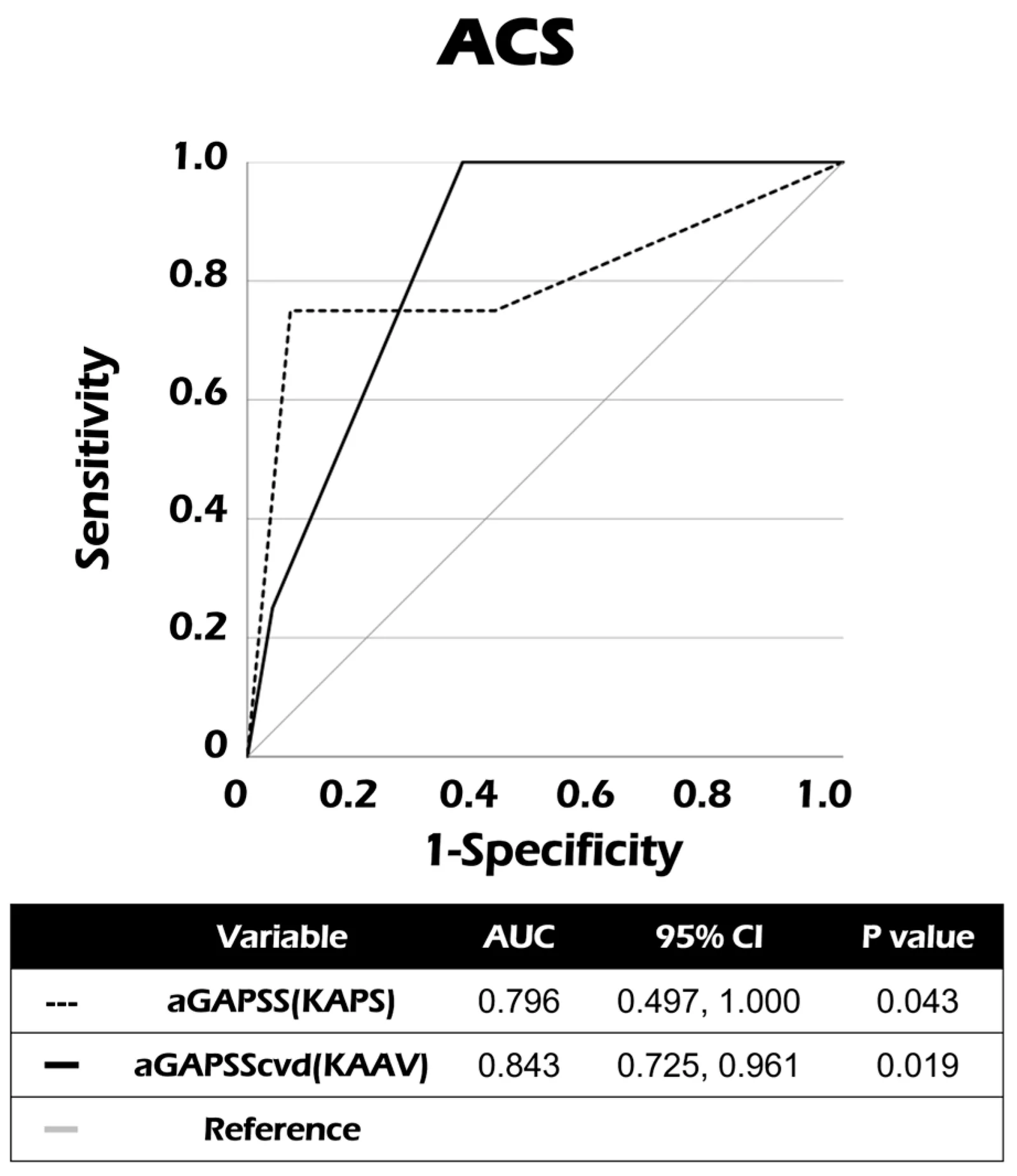
Accuracy of the aGAPSScvd (KAAV) in predicting ACS. ACS: Acute coronary syndrome; AUC: area under the curve; CI: confidence interval; aGAPSS (KAPS): adjusted global antiphospholipid syndrome score for Korean patients with antiphospholipid syndrome; aGAPSScvd (KAAV): adjusted global antiphospholipid syndrome score for ACS in Korean patients with antineutrophil cytoplasmic antibody-associated vasculitis.

The AUC of aGAPSScvd (KAAV) for ACS was 0.843 (95% CI: 0.725–0.961, *p* = 0.019), which was numerically larger than that of aGAPSS (KAPS) (AUC: 0.796, 95% CI: 0.497–1.000, *p* = 0.043) (Figure 2). In bootstrap internal validation with 1000 resamples, the estimated mean optimism in the AUC of the aGAPSScvd (KAAV) for predicting ACS was 0.006, resulting in an optimism-corrected AUC of 0.837.

**Figure 3:**
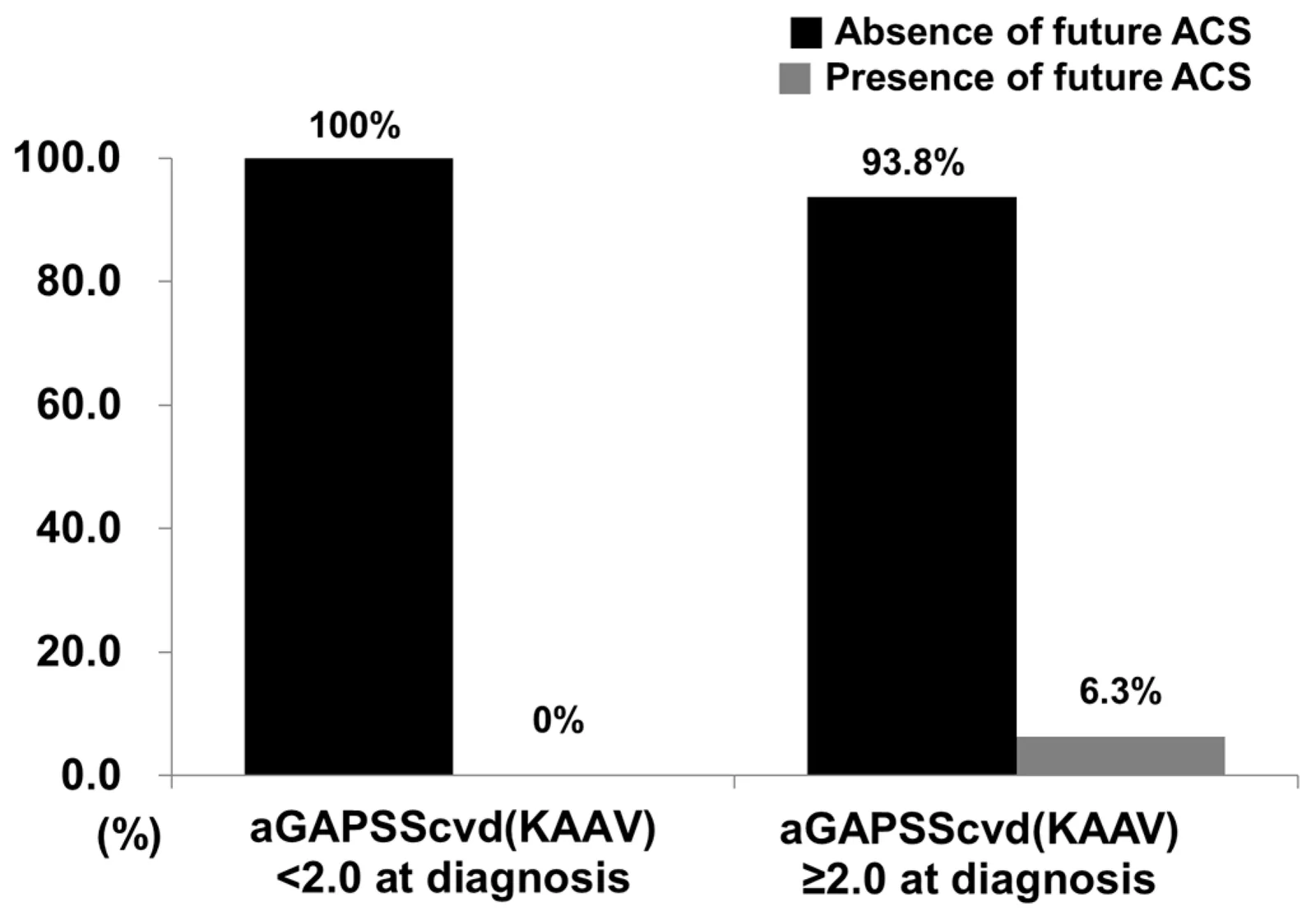
Proportion of patients developing ACS among those who had aGAPSScvd (KAAV) <2.0 and ≥2.0 at AAV diagnosis. ACS: Acute coronary syndrome; aGAPSScvd (KAAV): adjusted global antiphospholipid syndrome score for ACS in Korean patients with antineutrophil cytoplasmic antibody-associated vasculitis.

The cut-off value of aGAPSScvd (KAAV) that best predicted ACS was two with a sensitivity and specificity of 100% and 63.9%, respectively. Notably, none of the patients who had aGAPSScvd (KAAV) <2 at diagnosis developed ACS during follow-up (Figure 3).

**Figure 4:**
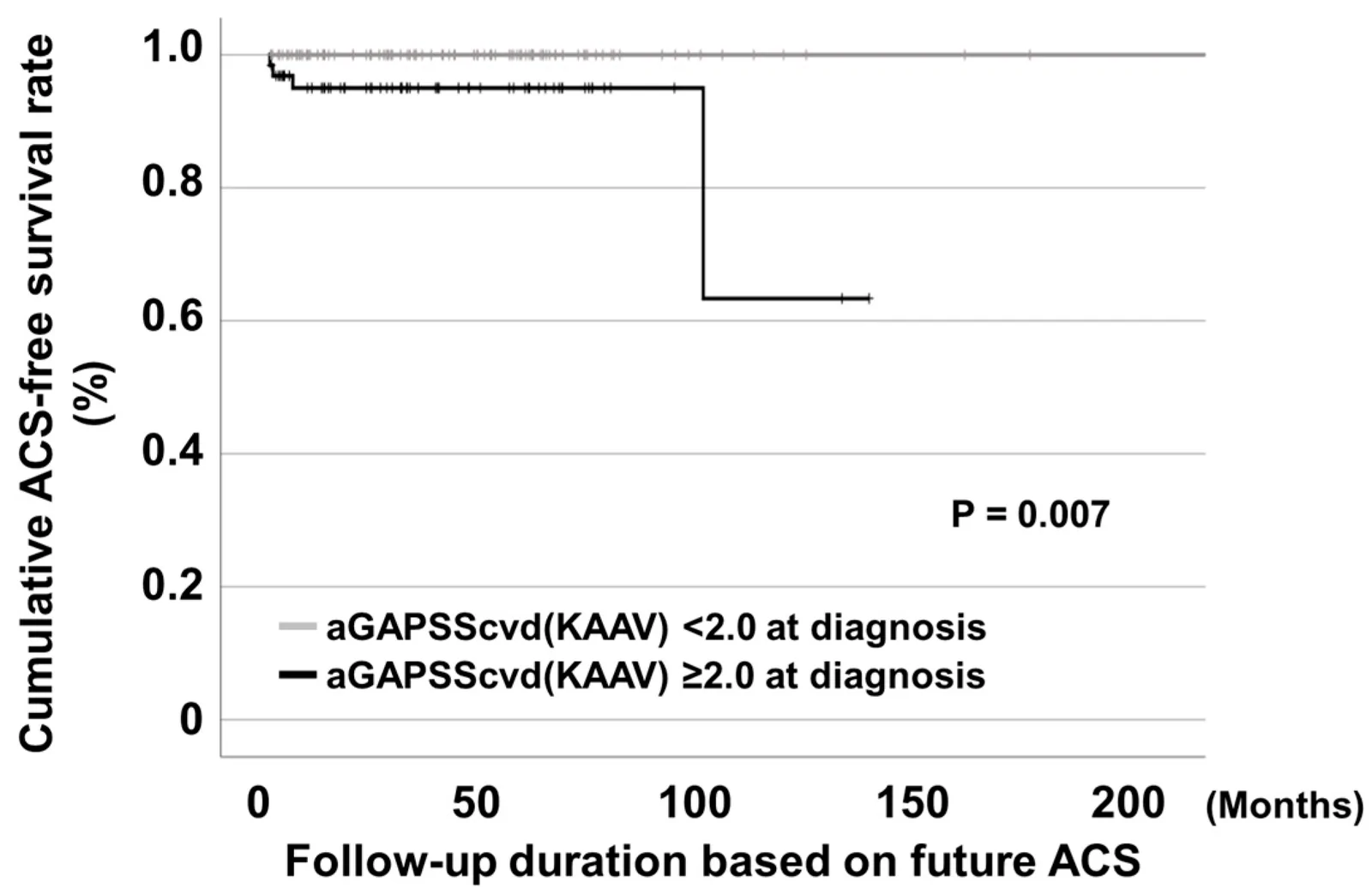
Comparison of ACS-free survival rate between patients with aGAPSScvd (KAAV) <2.0 and ≥2.0 at AAV diagnosis. ACS: Acute coronary syndrome; aGAPSScvd (KAAV): adjusted global antiphospholipid syndrome score for ACS in Korean patients with antineutrophil cytoplasmic antibody-associated vasculitis.

Moreover, patients who had aGAPSScvd (KAAV) <2 at diagnosis had a significantly higher ACS-free survival rate than those who had aGAPSScvd (KAAV) ≥2 at diagnosis (*p* = 0.007) (Figure 4).

## 4. Discussion

In this study, we assessed the accuracy of the previous risk score, aGAPSS, aGAPSScvd and aGAPSS (KAPS) on predicting cardiovascular diseases in Korean patients with AAV and found that only aGAPSS (KAPS) was statistically significant in predicting ACS. Moreover, an exploratory analysis yielded a two-item candidate index (aGAPSScvd [KAAV]) comprising LA and obesity. The candidate index showed an AUC of 0.843 for ACS. Patients with aGAPSScvd (KAAV) <2 at diagnosis had a higher ACS-free survival rate than those who had aGAPSScvd (KAAV) ≥2 at diagnosis. Notably, the cut-off value of two showed a sensitivity of 100% and a specificity of 63.9%. The high sensitivity suggests potential utility of this threshold as a screening tool for identifying patients at low risk of ACS, whereas its moderate specificity limits its usefulness as a rule-in threshold for identifying patients at high risk of ACS. Nevertheless, given the very small number of ACS events and the exploratory nature of the present analysis, the aGAPSScvd (KAAV) should be regarded as exploratory and hypothesis-generating rather than as a validated risk prediction model, and its proposed cut-off requires independent validation before clinical application.

In our Korean patients with AAV, aGAPSS (KAPS) (AUC: 0.796, 95% CI: 0.497–1.000, *p* = 0.043), but not aGAPSScvd (AUC: 0.752, 95% CI: 0.570–0.933, *p* = 0.086), was statistically significant in predicting ACS. Considering that aGAPSScvd is a scoring system developed specifically for detecting cardiovascular diseases [13], our finding is striking. The following point could be considered as a possible explanation for our finding. The underlying autoimmune diseases in the study populations in which the aGAPSScvd [13] and aGAPSS (KAPS) [15] were developed are different. The aGAPSScvd was developed in a study population where approximately half of the patients did not have any autoimmune diseases [13]. In contrast, all patients in the study population in which the aGAPSS (KAPS) was developed had autoimmune diseases (APS) [15]. Although APS and AAV are different diseases, they are in common in that they are both autoimmune diseases, and co-existence of APS and systemic vasculitis have been reported [23]. Therefore, the aGAPSS (KAPS) could be favourable in predicting ACS in patients with AAV than the aGAPSScvd.

The accuracy of the previous risk score in predicting ACS was poor with an AUC of 0.455 (95% CI: 0.200–0.710, *p* = 0.758) in our study population. The previous study has reported that the 5-year risk of cardiovascular diseases according to the risk score is as follows: risk score from −4.2 to −2.2, 5-year risk from 1.4 to 10%; risk score from −2.2 to −1.4, 5-year risk from 10 to 20%; and risk score from −1.4 to 0.29, 5-year risk from 20 to 43% [8]. The range of the previous risk score was somewhat narrow in our study population with an interquartile range of −1.95 to −0.93. The narrow distribution of the previous risk score could have resulted in an attenuated accuracy in our study population.

The aGAPSS (KAPS) was significant in predicting ACS, but not CVA. This could be explained by the different relative risks (RRs) of ACS and CVA in patients with AAV [24]. A meta-analysis on observational studies comparing patients with AAV and controls has reported that the RR of ischemic heart disease was 1.60 (95% CI: 1.39–1.84), whereas the RR of CVA was only 1.20 (95% CI: 0.98–1.48) [24], that is, the RR of CVA was lower than that of ischemic heart disease. As the RR of CVA is relatively lower, we assume that the discriminating ability of the scoring system could be weakened in predicting CVA, than in predicting ACS.

In our study population, the prevalence of aPLs was 9.4%, 5.3%, and 22.9% for aCL IgG/IgM, aβ2GPI IgG/IgM, and LA, respectively. The prevalence is similar to previous studies [4,5]. A study from the US has reported a prevalence of 12% and 2.8% for aCL IgG/IgM and aβ2GPI IgG/IgM, respectively in patients with GPA [4]. Another study from the UK has shown that the prevalence of LA or aCL IgG/IgM in patients with primary systemic vasculitis (majority of the patients had AAV) was 17.4% [5]. The similar prevalence of aPLs with the previous reports reflects external validity of our study cohort. In the multivariable Cox proportional hazards model, LA was included in the final model, whereas aCL IgG/IgM and aβ2GPI IgG/IgM were excluded. Considering that LA confers the highest risk of thrombosis among the aPLs [14,25], the inclusion of LA in the final model seems compelling.

Of the traditional cardiovascular risk factors, such as hyperlipidaemia, hypertension, T2DM, obesity, and smoking history [26], only obesity was included as a component in the aGAPSScvd (KAAV). Obesity not only contributes to the occurrence of cardiovascular diseases independent of the other traditional cardiovascular risk factors, but also leads to the occurrence of other traditional cardiovascular risk factors, including hyperlipidaemia, hypertension, and T2DM [27]. Therefore, the inclusion of obesity, instead of other traditional cardiovascular risk factors, appears reasonable.

The cut-off value of two showed a high sensitivity of 100% but a moderate specificity of 63.9%. Although this finding suggests that the candidate index may be useful for identifying patients at low risk of ACS, its moderate specificity suggests limited ability to identify patients at higher risk. Accordingly, a score <2 may indicate a lower risk of ACS, whereas a score ≥2 should not be considered an indication for automatic cardiology referral or coronary angiography. Rather, a score ≥2 may warrant greater attention to cardiovascular risk and closer observation for the development of cardiovascular symptoms. However, given the exploratory nature of the analysis, the very small number of ACS events, and the lack of external validation, the present findings are insufficient to support a specific cardiovascular investigation or referral strategy based solely on the candidate index. Further validation is required before the aGAPSScvd (KAAV) can be incorporated into clinical decision-making for cardiovascular risk assessment. Additional cardiovascular and disease-related factors, such as more detailed metabolic parameters, inflammatory burden, and renal function, may potentially improve the specificity and overall discriminatory performance of the candidate index. However, given the very small number of ACS events in the present study, the inclusion of additional predictors was not feasible without substantially increasing the risk of model instability and overfitting. This issue should be further investigated in larger studies with a sufficient number of ACS events.

There are some limitations in our study. First, only four ACS events occurred during follow-up. This extremely small number of events substantially limits the stability and precision of the multivariable model and may have resulted in unstable coefficient estimates, data-driven variable selection, overfitting, and overestimation of the apparent discriminatory performance. Although bootstrap internal validation was performed to estimate optimism, this approach cannot overcome the fundamental limitation imposed by the small number of outcome events. Accordingly, the present analysis should not be interpreted as the development or validation of a clinical risk prediction model; rather, the aGAPSScvd (KAAV) should be regarded only as an exploratory, hypothesis-generating candidate index. Moreover, the derived coefficients and the proposed threshold should be considered preliminary, particularly because the threshold was selected and evaluated in the same cohort. Larger studies are needed to validate our finding. Second, medications used during follow-up may have affected the risk of ACS and therefore represent a potential source of residual confounding. However, medication use was not incorporated into the candidate index because the index was intended to be based on covariates available at the time of AAV diagnosis. Third, anthropometric indices, such as a body shape index, lipid accumulation product, visceral adiposity index, and body roundness index, could not be calculated because the required anthropometric measurements were not available in our retrospective dataset. Future prospective studies incorporating detailed anthropometric measurements are needed to determine whether these indices could further improve cardiovascular risk assessment in patients with AAV. Fourth, this was a single-centre study including only Korean patients, which limits the generalisability of our findings. There are disparities in characteristics of AAV across different ethnic populations [28]. Therefore, the present findings should not be directly extrapolated to other ethnic populations, and further external validation in independent, multicentre and multiethnic cohorts is needed.

## 5. Conclusions

Among the derivatives of aGAPSS, we found that aGAPSS (KAPS) was statistically significant in predicting ACS in patients with AAV. In addition, an exploratory two-item candidate index (aGAPSScvd [KAAV]) comprising LA and obesity showed discriminatory performance for ACS. Given the exploratory nature of the present analysis and the very small number of ACS events, validation of this candidate index in larger independent multicentre cohorts with an adequate number of events is required.

## Figures and Tables

**Table 1:** Characteristics of patients with AAV (N = 170).

Variables	Values
***At the time of AAV diagnosis***	
**General data**	
Age (years)	63.0 (50.0–70.3)
Male sex (N, (%))	67 (39.4)
Body mass index (kg/m^2^)	22.7 (20.3–24.2)
Smoking history (N, (%))	9 (5.3)
**AAV Subtypes (N, (%))**	
MPA	93 (54.7)
GPA	44 (25.9)
EGPA	33 (19.4)
**ANCA positivity**	
MPO-ANCA	124 (72.9)
PR3-ANCA	26 (15.3)
**Items of modified aGAPSScvd**	
aCL IgG/IgM	16 (9.4)
aβ2GPI IgG/IgM	9 (5.3)
LA	39 (22.9)
Hyperlipidaemia	31 (18.2)
Hypertension	62 (36.5)
T2DM	41 (24.1)
Obesity	33 (19.4)
Smoking history	9 (5.3)
**Previous risk score**	−1.26 (−1.95–−0.93)
**aGAPSS**	3.0 (0–4.0)
**aGAPSScvd**	3.0 (0–6.0)
**aGAPSS (KAPS)**	0 (0–1.0)
***During the follow-up period***	
**Poor outcomes during follow-up (N, (%))**	
CVA (N, (%))	17 (10.0)
Follow-up duration based on CVA (months)	32.1 (10.6–59.2)
ACS (N, (%))	4 (2.4)
Follow-up duration based on ACS (months)	35.4 (12.4–61.2)
**Medications administered during follow-up (N, (%))**	
Glucocorticoid	168 (98.8)
Cyclophosphamide	102 (60.0)
Rituximab	34 (20.0)
Azathioprine	98 (57.6)
Mycophenolate mofetil	43 (25.3)
Tacrolimus	13 (7.6)
Methotrexate	11 (6.5)

Values are expressed as a median (Q1–Q3) or number (percentage). AAV: ANCA-associated vasculitis; ANCA: antineutrophil cytoplasmic antibody; MPA: microscopic polyangiitis; GPA: granulomatosis with polyangiitis; EGPA: eosinophilic GPA; MPO: myeloperoxidase; PR3: proteinase 3; aGAPSS: adjusted global antiphospholipid syndrome score; CVD: cardiovascular disease; KAPS: Korean patients with antiphospholipid syndrome; aCLs: anticardiolipin antibodies; aβ2GPIs: anti-beta2 glycoprotein I antibodies; LA: lupus anticoagulant; T2DM: type 2 diabetes mellitus; CVA: cerebrovascular accident; ACS: acute coronary syndrome.

**Table 2:** Cox proportional hazards model analyses of each parameter composing of aGAPSScvd at diagnosis for ACS during follow-up in patients with AAV (N = 170).

Variables	Univariable			Multivariable			
	HR	95% CI	*p* Value	B	HR	95% CI	*p* Value
Step 1							
aCL IgG/IgM	0.043	0.000, 1,726,838.597	0.043				
aβ2GPI IgG/IgM	0.040	0.000, 168,952.334	0.679				
LA	8.968	0.926, 86.553	0.058		6.851	0.695, 67.527	0.099
Hyperlipidaemia	0.036	0.000, 2011.243	0.552				
Hypertension	0.438	0.044, 4.405	0.484				
T2DM	7.584	0.760, 75.674	0.084		4.382	0.425, 45.201	0.215
Obesity	9.209	0.831, 101.995	0.070		7.231	0.638, 81.948	0.110
Smoking history	0.044	0.000, 1,879,062.894	0.728				
Step 2							
LA				2.152	8.603	0.892, 82.986	0.063
Obesity				2.165	8.713	0.786, 96.525	0.078

aGAPSS: Adjusted global antiphospholipid syndrome score; CVD: cardiovascular disease; AAV: ANCA-associated vasculitis; ANCA: antineutrophil cytoplasmic antibody; HR: hazard ratio; CI: confidence interval; aCLs: anticardiolipin antibodies; aβ2GPIs: anti-beta2 glycoprotein I antibodies; LA: lupus anticoagulant; T2DM: type 2 diabetes mellitus. Candidate index based on aGAPSScvd for ACS in patients with AAV (aGAPSScvd (KAAV)) = 2 × LA + 2 × obesity.

## Data Availability

The data that support the findings of this article are available on request from the corresponding author.

## Abbreviations

AAV	antineutrophil cytoplasmic antibody-associated vasculitis
aβ2GPI	anti-β2 glycoprotein I
aCL	anticardiolipin
ACS	acute coronary syndrome
aGAPSS	the adjusted global antiphospholipid syndrome score
aGAPSScvd	the adjusted global antiphospholipid syndrome score for cardiovascular diseases
aGAPSScvd [KAAV]	the adjusted global antiphospholipid syndrome score for cardiovascular diseases for Korean patients with AAV
aGAPSS [KAPS]	the adjusted global antiphospholipid syndrome score for Korean patients with APS
ANCA	antineutrophil cytoplasmic antibody
Anti-PS/PT	anti-phosphatidylserine/prothrombin
aPLs	antiphospholipid antibodies
APS	antiphospholipid syndrome
AUC	the area under the curve
BMI	body mass index
BVAS	Birmingham Vasculitis Activity score
CI	confidence interval
CVA	cerebrovascular accident
EGPA	eosinophilic granulomatosis with polyangiitis
GAPSS	the global antiphospholipid syndrome score
GPA	granulomatosis with polyangiitis
Ig	immunoglobulin
IRB	the Institutional Review Board
LA	lupus anticoagulant
MPA	microscopic polyangiitis
MPO	myeloperoxidase
PR3	proteinase 3
RR	relative risk
T2DM	type 2 diabetes mellitus
